# Correction: TAK1 inhibition by natural cyclopeptide RA-V promotes apoptosis and inhibits protective autophagy in Kras-dependent non-small-cell lung carcinoma cells

**DOI:** 10.1039/d5ra90088c

**Published:** 2025-07-14

**Authors:** Jianhong Yang, Tao Yang, Wei Yan, Dan Li, Fang Wang, Zhe Wang, Yingjie Guo, Peng Bai, Ninghua Tan, Lijuan Chen

**Affiliations:** a Cancer Center, West China Hospital, Sichuan University, Collaborative Innovation Center for Biotherapy Chengdu China chenlijuan125@163.com; b School of Traditional Chinese Pharmacy, State Key Laboratory of Natural Medicines, China Pharmaceutical University Nanjing China nhtan@cpu.edu.cn; c Department of Cardiology, The Second Affiliated Hospital of Guangdong Medical University Zhanjiang Guangdong China

## Abstract

Correction for ‘TAK1 inhibition by natural cyclopeptide RA-V promotes apoptosis and inhibits protective autophagy in Kras-dependent non-small-cell lung carcinoma cells’ by Jianhong Yang *et al.*, *RSC Adv*., 2018, **8**, 23451–23458, https://doi.org/10.1039/C8RA04241A.

The authors regret an error in Fig. 4b where an incorrect β-actin band was used for the H441 cells. The corrected Fig. 4 is shown below.

**Fig. 4:**
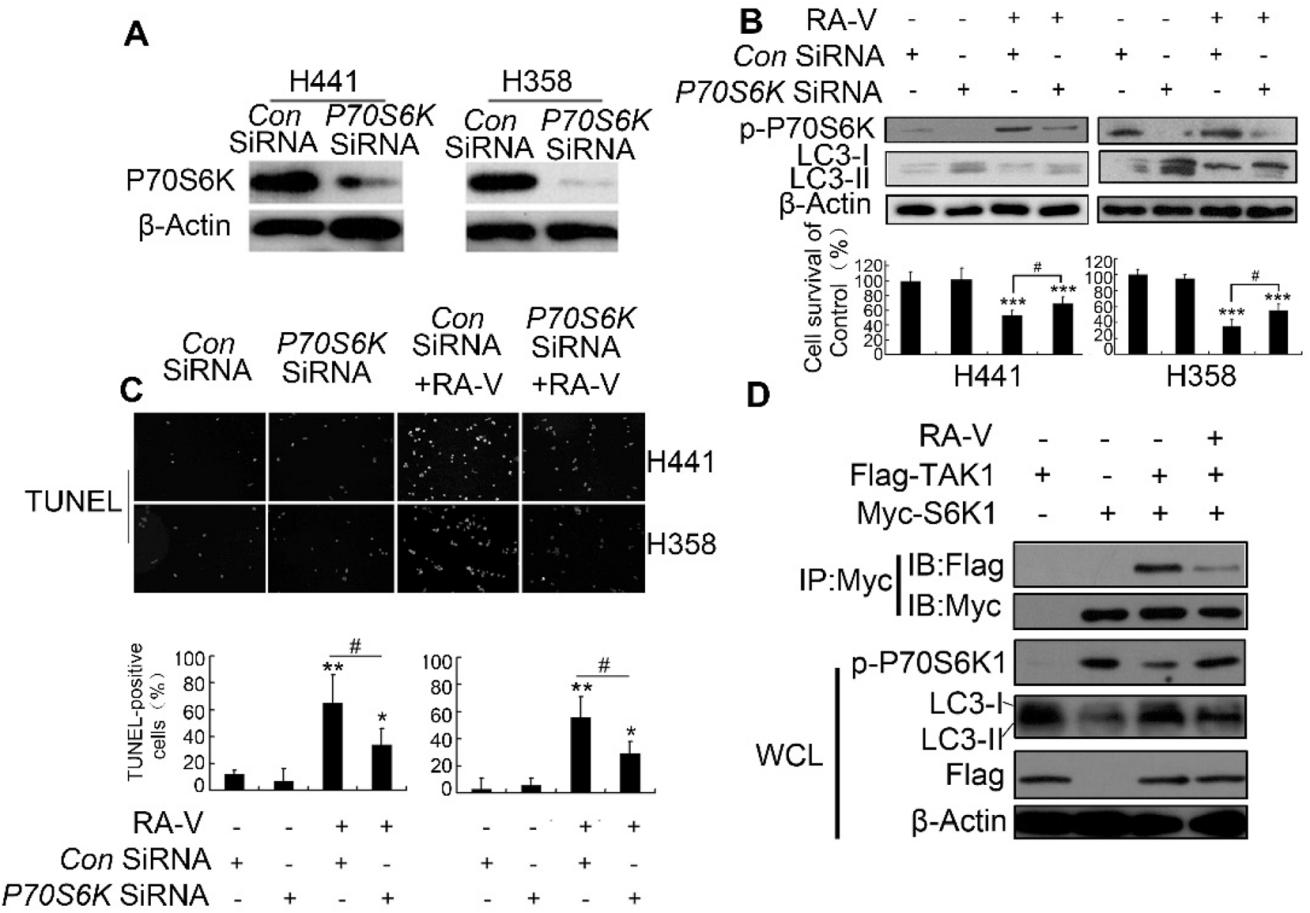
Inhibition of the TAK1/P70S6K pathways helps to protective basal autophagy inhibition by RA-V. (A) Detection of SiRNA effects: detection of P70S6K expression by western blotting in P70S6K-knocked-down cells. (B) RA-V were treated with or without control or *P70S6K*-silenced H358 or H441 cells for 48 h, cell viability was detected by MTT, and the expression of p-P70S6K and LC3 were detected by western blotting. (C) RA-V were treated with or without control or *P70S6K*-silenced H358 or H441 cells for 48 h, and apoptosis was detected by TUNEL assay. (D) H293T cells transfected with Flag-tagged TAK1 construct and Myc-tagged S6K1 construct alone or in combination, then treated with or without 100 nM of RA-V. Cell lysates were collected and immunoblotted with an anti-myc-antibody, followed by immunoblotting with flag or myc. The blots in the lower four panels were obtained from the same cell lysates using the indicated antibodies. Data are presented as means ± standard deviation. *P < 0.05, **P < 0.01, ***P < 0.01, as compared to controls or the appointed group.

The authors confirm that this correction does not affect the original conclusions of the study.

The Royal Society of Chemistry apologises for these errors and any consequent inconvenience to authors and readers.

